# Abnormal Distribution of Nucleic Acid in Tissue Culture Cells Infected with Polyoma Virus

**DOI:** 10.1038/bjc.1960.37

**Published:** 1960-06

**Authors:** A. C. Allison, J. A. Armstrong

## Abstract

**Images:**


### ABNORMAL DISTRIBUTION OF NUCLEIC ACID IN TISSUE CULTURE CELLS INFECTED WITH POLYOMA VIRUS

A. C. ALLISON AND J. A. ARMSTRONG

*From the National Institute for Medical Research, Mill Hill, London, N.W.7*

Received for publication April 13, 1960

THE polyoma virus, originally isolated from mice, has been shown to produce multiple tumours when inoculated into new-born mice and hamsters (Stewart, Eddy and Borgese, 1958). Growth of the virus in mouse embryo tissue cultures is accompanied by a regular cytopathic effect, notably clumping of cells and detachment from glass surfaces (Eddy, Stewart and Berkeley, 1958). The fluorescent antibody technique has shown that in polyoma-infected tissue cultures of mouse embryo cells viral antigenic material develops first in the host cell nuclei and appears later in the cytoplasm (Henle, Deinhart and Rodriguez, 1959). Electron micrographs of ultrathin sections of tissue culture cells infected with the prototype SE and Mill Hill strains of polyoma virus have shown large numbers of virus-like particles, mostly in the nuclei (Bernhard, Febvre and Cramer, 1959; Dmochowski, Grey and Magee, 1959; Negroni, Dourmashkin and Chesterman, 1959).

Evidence concerning the nucleic acid component of polyoma virus has been conflicting. Cytochemical changes in infected mouse lymphoma cells were reported by Love and Rabson (1959) who used the toluidine blue–molybdate and Feulgen techniques. Cellular enlargement was associated with the appearance of abnormal nuclear vacuoles which developed a staining reaction for ribonucleoprotein. From these findings, it was inferred that normal nuclear mechanisms of ribonucleoprotein synthesis may be diverted into the process of virus replication. No evidence of an increased synthesis of deoxyribonucleic acid (DNA) was found. On the other hand, Di Mayorca *et al.* (1959) have very recently reported that virus-free nucleic acid extracts of polyoma-infected tissue cultures could reproduce the typical infection in other tissue cultures. The infectivity of the extracts, unlike that of intact virus, was abolished by incubation with deoxyribonuclease. Ribonuclease had no effect, and it was concluded that polyoma is probably a DNA virus.

In view of this discrepancy it may be helpful to record briefly some observations recently made in this laboratory which are at variance with the previously published cytochemical data. The technique employed has been the acridine orange polychromatic fluorescence technique for differentiation of nucleic acids, which is particularly suited to the study of virus cytopathology in tissue cultures (Armstrong, 1956; Armstrong and Niven, 1957).

### MATERIALS AND METHODS

### *Cells*

Three cell types were used. New-born mouse kidney and mouse embryo cell cultures were prepared as described by Rowe *et al.* (1959). They were grown for four days as stationary cultures in flat 4-ounce bottles in the following medium :

Gey's saline, 80 parts ; lactalbumin hydrolysate (5 per cent of stock) 10 parts ; calf serum, 10 parts ; penicillin 100 units per ml. ; streptomycin 100 $\mu$g. per ml. ; and Mycostatin 50 units per ml. The cells were trypsinized, washed and transferred in 2 ml. volumes containing $10^6$ cells into test-tubes with coverslips. After 3 days' growth in the same nutrient medium, the medium was replaced with another containing 5 per cent calf serum and the virus inoculum. A strain of "L" cells (obtained from Dr. J. C. N. Westwood, Microbiological Research Establishment, Porton, Wilts.) was maintained in a medium consisting of Gey's saline 70 parts ; calf serum, 20 parts ; yeast extract (1 per cent of stock) 10 parts ; lactalbumin hydrolysate (5 per cent of stock) 10 parts ; and antibiotics as above. Trypsinized, washed cells were transferred to test tubes containing coverslips, grown and infected in a medium containing 10 parts of calf serum.

*Virus*

SE polyoma virus, batch 3E29–4, obtained from Dr. B. E. Eddy, National Cancer Institute, Bethesda, Md., U.S.A., was propagated in mouse embryo cell cultures in flat bottles. After 7 days' incubation with two changes of medium the cells were removed and treated with fluorocarbon (Giardi, 1959) to prepare stock virus. Virus was titrated by haemagglutination of guinea-pig cells and infectivity in mouse enbryo tissue cultures (Rowe *et al.*, 1959).

*Microscopy*

Coverslips from control and infected cultures were removed at daily intervals up to 8 days after virus inoculation, rinsed in tris-buffered Gey's solution (pH 7·4) and fixed in absolute alcohol. The method of staining coverslip cultures with 0·05 per cent acridine orange (C.I. 788) in acetate-HCl buffer (pH 2·7) was described by Armstrong and Hopper (1959). Examination was by dark-field fluorescence microscopy, using the blue-violet emission of a high pressure mercury vapour lamp for activation of fluorescence. Other cultures were fixed for 20 minutes in 1 per cent osmium tetroxide buffered to pH 7·4 (Palade, 1952) ; after rinsing in Gey's solution they were allowed to stand overnight in 70 per cent ethanol and finally mounted in water for examination by phase-contrast microscopy.

### OBSERVATIONS

*Virus yield*

The appearance of infective virus in mouse kidney cells and culture fluid after inoculation with $10^5$ $TCD_{50}$ of polyoma virus is shown in Fig. 1. Very little virus could be recovered at 24 hours. There was a considerable increase of cell-associated virus between the second and third days after infection, with very little virus liberation into the culture medium. From the third to the eighth day after infection there was a slower increase in cell-associated virus and some increase in the virus liberated into the medium. The latter, however, was never more than one-tenth of the total virus in the preparation. In cultures of "L" cells virus yield was low at all times and only a small proportion of cells showed morphological changes.

*Cytopathic effect*

Unstained cultures of the mouse kidney and mouse embryo cells examined under the low power of the microscope showed no change until the third day after infection, when some cells appeared to be clumped, rounded and refractile. This appearance increased daily, with progressive detachment of cells into the culture medium. But on the eighth day after infection, when disintegration of the cell sheet was sufficient to be visible to the naked eye, large intervening areas containing apparently normal cells were always still present. The cultures of " L " cells showed less marked but consistent cytopathic effects between the fifth and eighth days after infection.

FIG. 1.—Appearance of cell-associated virus (continuous line) and virus in medium (interrupted line) in mouse kidney cultures infected with polyoma virus.

*Cytology*

The fluorescence of cells in uninfected mouse kidney cell cultures after treatment with acridine orange is reproduced in black and white in Fig. 2. In actual specimens the nuclear margin and chromatin network (including nucleolus-associated chromatin) emitted the greenish-yellow fluorescence which is typical of DNA-containing elements. This contrasted sharply with the orange-red colour of the nucleoli themselves, and of the ribonucleoprotein which is scattered throughout the cytoplasm.

No distinctive cytological changes were observed in these cultures up to 3 days after inoculation with polyoma virus, but on the fourth day there were multiple foci in which cells had become rounded and were easily detached from the glass surface. From this time up to the eighth day of infection an unusual type of intranuclear structure was detectable in many of the cells close to where rounding up and cell detachment were in progress. It was found only occasionally in cells of the intervening, and intact, parts of the cell sheet. The material concerned gave an intense greenish-yellow fluorescence of the DNA type. In 4 day-infected cultures it was mostly located in one or more discrete centres within the nucleoplasm ; but by the eighth day a high percentage of the affected nuclei were almost completely filled. Detection of the earliest stage was made difficult by the presence of rather large chromatin granules in the normal cell nuclei, this being a constant feature of many uninfected murine cells in tissue culture (Fig. 2) ; some nuclei showing minimal alteration of the DNA pattern after infection may have been overlooked on this account. Nuclear fragmentation was rarely seen in spite of progressive accumulation of the abnormal intranuclear material. As illustrated in Fig. 3–6, the appearances were very different from those of a non-specific degeneration or of pyknosis. The figures show four typically affected nuclei arranged in order of increasing abnormality ; the black and white prints were prepared from original colour transparencies. The positions of nucleoli, identifiable because of the red fluorescence of their RNA, are indicated as a guide in Fig. 2 and 4 ; the remaining nuclear fluorescence was entirely of the DNA type. In addition to nuclear changes there was vacuolation of the cytoplasm in some of these cells (Fig. 3 and 6). No regular change was noted in the structure of the nucleoli or in their fluorescent intensity. None of the infected mouse kidney cell cultures showed evidence of DNA-containing material in the cytoplasm.

Almost identical changes were found in the polyoma-infected mouse embryo cultures ; and a few scattered cells with the same abnormal patterns of intranuclear DNA were present, in the corresponding cultures of " L " strain cells. In the latter, however, occasional cells with abnormal nuclei had in their cytoplasm a prominent paranuclear mass of greenish-yellow fluorescent material (Fig. 7). This was quite distinct from the dull green fluorescence sometimes found in a hypertrophied Golgi zone of cultured epithelial cells. It was also the only indication of a cytoplasmic DNA reaction in the polyoma-infected cultures at our disposal.

Phase-contrast study of the osmium-fixed kidney cell and mouse embryo cultures confirmed that nuclear changes of a peculiar kind occurred in the vicinity of the focal cytopathic effect. It has been shown that osmium fixation avoids structural artefacts which are sometimes brought about by the moie commonly used histological fixatives (Strangeways and Canti, 1927). Moreover, cytological details may be revealed by phase-contrast with greater clarity than is usually possible in the equivalent living cell. A normal cell nucleus is shown in Fig. 8 ; the nucleoli are very dense relative to the surrounding nucleoplasm, which is faintly stippled. The type of nucleus seen in Fig. 9, however, occurred only after the fourth day of infection with polyoma virus. Irregular patches of somewhat lower density than the nucleoli, and of varying size, are present in the nucleoplasm ; their appearance resembles closely that of the material revealed by fluorescence microscopy after alcohol fixation (Fig. 3 and 4). In view of this it seems likely that the distribution of nuclear contents shown by the fluorescence microscope was not, in this case, much influenced by fixation artefact.

---

EXPLANATION OF PLATES

FIG. 2–7 are fluorescence photomicrographs of cells in tissue culture, stained by the acridine orange technique. Prints were made from colour transparencies. The nucleoli (shown by arrows in Fig. 1 and 3) were red (RNA), the remaining nuclear fluorescence greenish-yellow (DNA).

FIG. 2.—Uninfected mouse kidney cells, × 1200.

FIG. 3–6.—Abnormal patterns of nuclear DNA fluorescence in mouse kidney cells, between 4 and 8 days after infection with polyoma virus, ×1200.

FIG. 7.—" L " strain cell showing a cytoplasmic mass of DNA-type fluorescence 6 days after infection with polyoma virus, ×1700.

FIG. 8.—Phase-contrast photomicrograph, showing the nucleus of a normal mouse kidney cell in tissue culture. $OsO_4$ fixation, ×1800.

FIG. 9.—Similar preparation to Fig. 8, but from a culture infected 8 days previously with polyoma virus. $OsO_4$ fixation, ×1800.

2

3

4

5

6

7

8

9

Allison and Armstrong.

### COMMENT

The cytochemical changes reported here are reminiscent of the abnormal patterns of intranuclear DNA which are found in human cell cultures infected with adenoviruses, and which are clearly demonstrated by the acridine orange technique (Armstrong and Hopper, 1959). However, with polyoma the DNA changes were not associated with the remarkable distortion of nuclear morphology which is so typical of adenovirus infections and which is greatly influenced by alcohol fixation. The present findings would appear, therefore, to be consistent with the replication of a DNA virus within the nuclei of susceptible murine cells. Close inspection of the control specimens revealed no comparable DNA pattern, so that the possibility of a spontaneous development of bizarre nuclear forms is very unlikely. We interpret the present findings in polyoma-infected cultures as additional evidence for the view, expressed by Di Mayorca *et al.* (1959), that the polyoma agent is in all probability a DNA virus.

The first indication of infection is the appearance of multiple foci of DNA-containing material in the nucleoplasm. The findings suggest that these enlarge, coalesce and eventually fill the nucleus. Cells at this stage of virus development presumably correspond to those which have shown large numbers of virus-like inclusions when examined by electron microscopy. In infected " L " cell cultures, a few cells were seen with DNA-containing material in the cytoplasm, suggesting a discharge of virus nucleic acid from the nucleus into the cytoplasm, but in the mouse kidney and mouse embryo cells there were no signs of cytoplasmic DNA. This might be related to the fact that moribund cells with large amounts of intranuclear virus are detached from the coverslips before disintegration and release of virus. Certainly the occurrence of recognizable cells in the medium coincided with the appearance in it of detectable virus. In general, the fluorescence induced by acridine orange staining is comparable with that obtained in the earlier stages of infection by means of the fluorescent antibody technique (Henle *et al.*, 1959), but with the latter some cells in the late cultures showed cytoplasmic fluorescence. This might be due to the passage of virus from the nucleus into the cytoplasm, but alternatively could indicate the presence in the cytoplasm of antigen not accompanied by nucleic acid and not therefore visualized by acridine orange.

A relatively long latent period and slow progression of infection with the polyoma virus appears to be the rule, and despite the use of relatively large virus inocula only a minority of cells showed cytopathic effects and accumulation of DNA-containing material in the nuclei. It is conceivable that other parts of the cell sheet were infected but transformed into tumour-like cells releasing very little virus as suggested recently by Vogt and Dulbecco (1960).

### SUMMARY

The progress of infection with polyoma virus in tissue cultures of mouse kidney cells, mouse embryo cells and " L " cells has been followed by titration of virus and the appearance of cytochemical changes.

Infective virus accumulates gradually and is slowly released from infected cells. In spite of large initial virus inocula only a minority of cells showed characteristic cytopathic effects.

After infection the first change detectable in cells stained with acridine orange and examined by fluorescence microscopy was the appearance of abnormal DNA-containing foci in the nuclei. At a later stage cells became detached from the coverslips and the nuclei of some were by then almost filled with the DNA-containing material. It is concluded that polyoma virus contains DNA and multiplies in the nuclei of susceptible cells.

We are indebted to Mr. M. R. Young for taking the photomicrographs.

### REFERENCES

ARMSTRONG, J. A.—(1956) *Exp. Cell. Res.*, **11**, 640.
*Idem* AND HOPPER, P. K.—(1959) *Ibid.*, **16**, 584.
*Idem* AND NIVEN, J. S. F.—(1957) *Nature, Lond.*, **180**, 1335.
BERNHARD, W., FEBVRE, H. L. AND CRAMER, R.—(1959) *C.R. Acad. Sci., Paris*, **249**, 483.
DI MAYORCA, G. A., EDDY, B. E., STEWART, S. E., HUNTER, W. S., FRIEND, C. AND BENDICH, A.—(1959) *Proc. nat. Acad. Sci., Wash.*, **45**, 1804.
DMOCHOWSKI, L., GREY, C. E. AND MAGEE, L. A.—(1959) *Proc. Soc. exp. Biol. N.Y.*, **102**, 575.
EDDY, B. E., STEWART, S. E. AND BERKELEY, W.—(1958) *Ibid.*, **98**, 848.
GIARDI, A. J.—(1959) *Virology*, **9**, 488.
HENLE, G., DEINHARDT, F. AND RODRIGUEZ, J.—(1959) *Ibid.*, **8**, 388.
LOVE, R. AND RABSON, A. S.—(1959) *J. nat. Cancer Inst.*, **23**, 875.
NEGRONI, G., DOURMASHKIN, R. AND CHESTERMAN, F. C.—(1959) *Brit. med. J.*, ii, 1359.
PALADE, G. E.—(1952) *J. exp. Med.*, **95**, 285.
ROWE, W. P., HARTLEY, J. W., ESTES, J. D. AND HUEBNER, R. J.—(1959) *Ibid.*, **109** 379.
STEWART, S. E., EDDY, B. E. AND BORGESE, N. G.—(1958) *J. nat. Cancer Inst.*, **20**, 1223.
STRANGEWAYS, T. S. P. AND CANTI, R. G.—(1927) *Quart. J. micr. Sci.*, **71**, 1.
VOGT, M. AND DULBECCO, R.—(1960) *Proc. nat. Acad. Sci. Wash.*, **46**, 365.


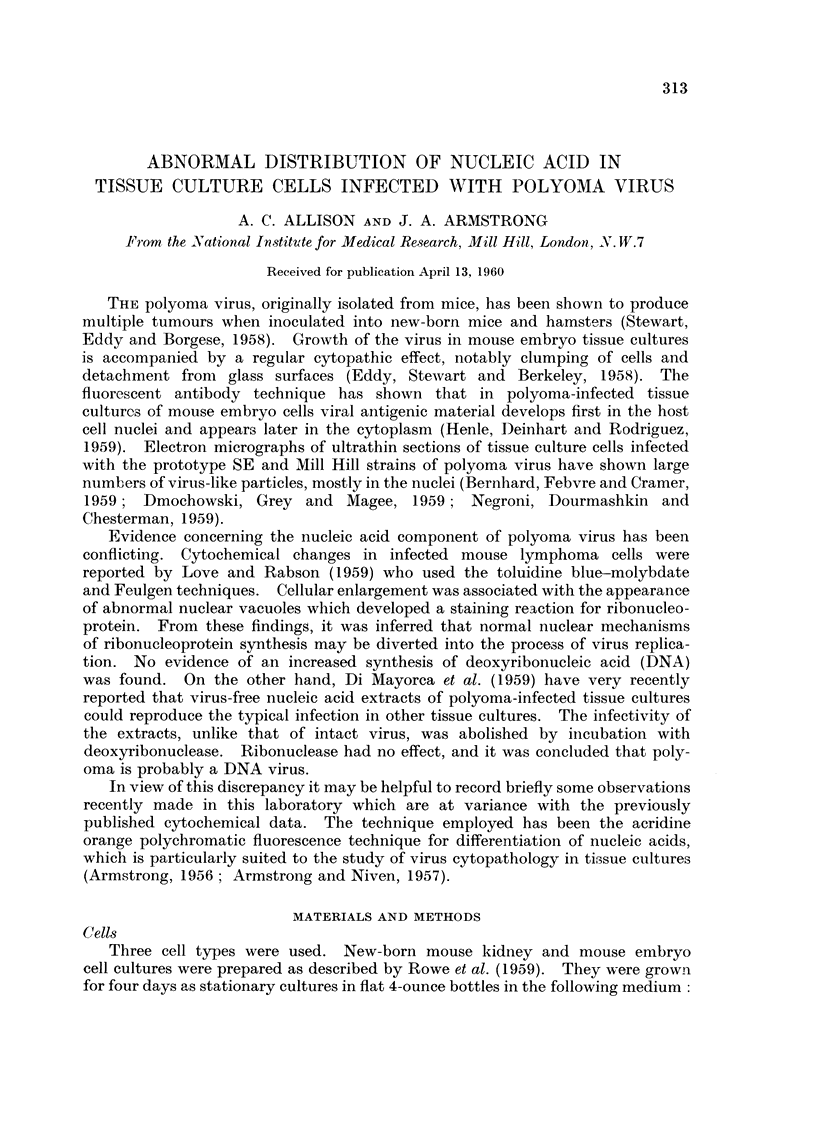

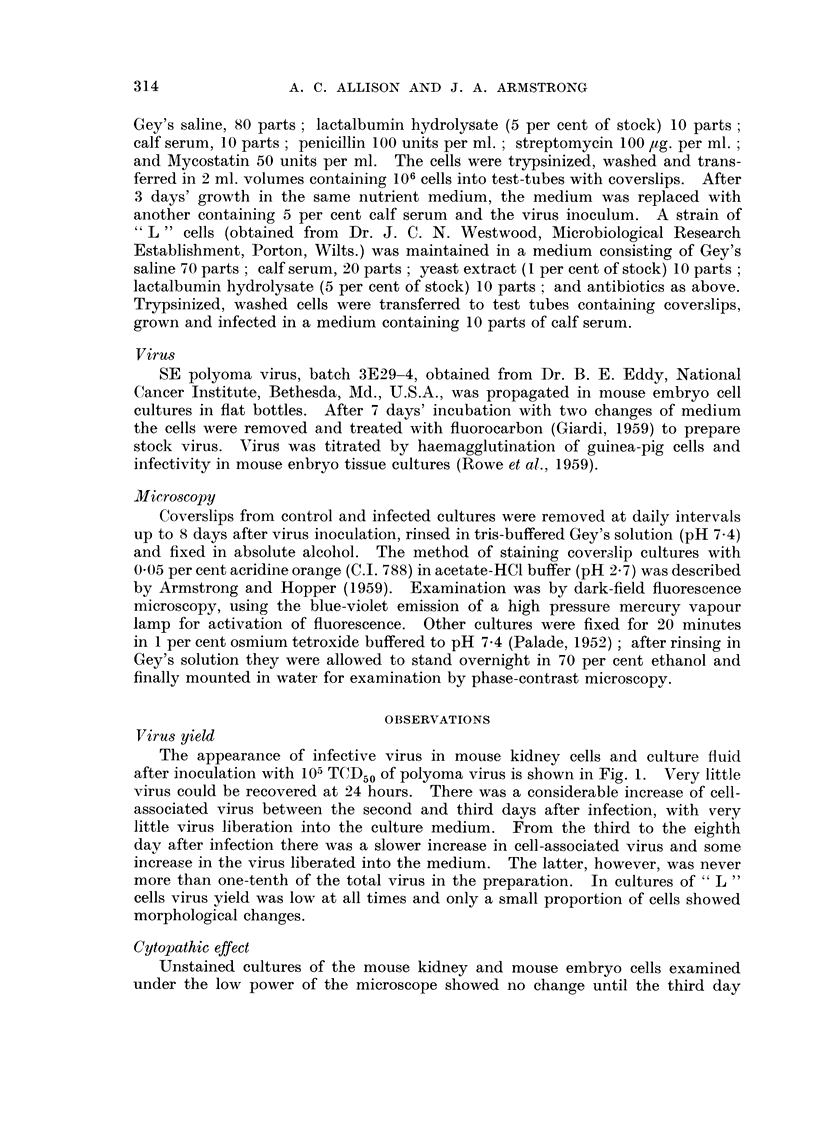

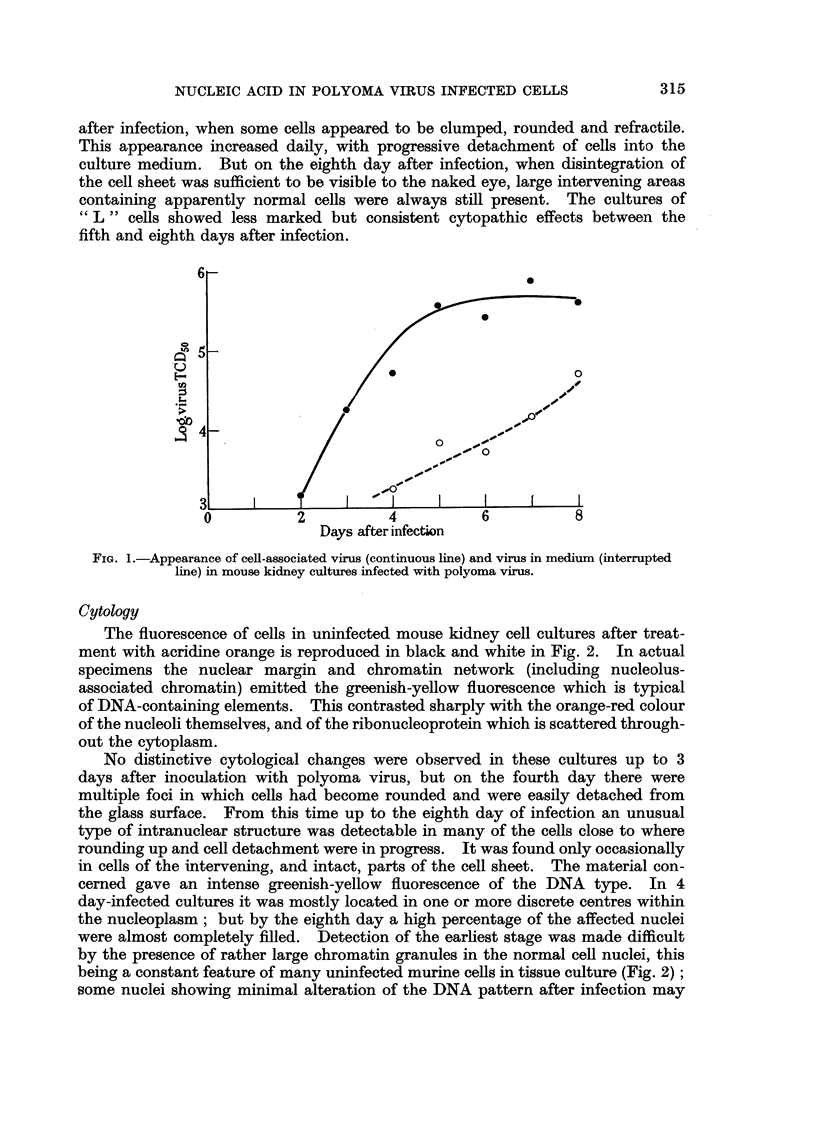

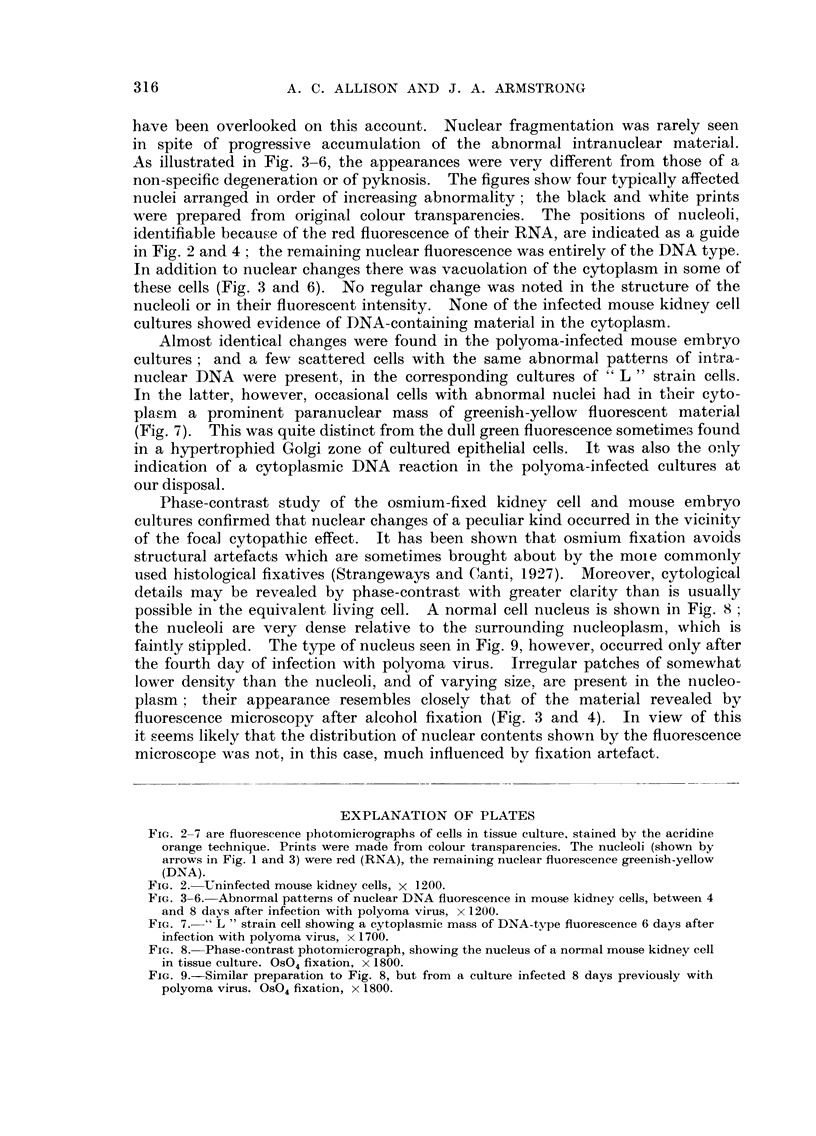

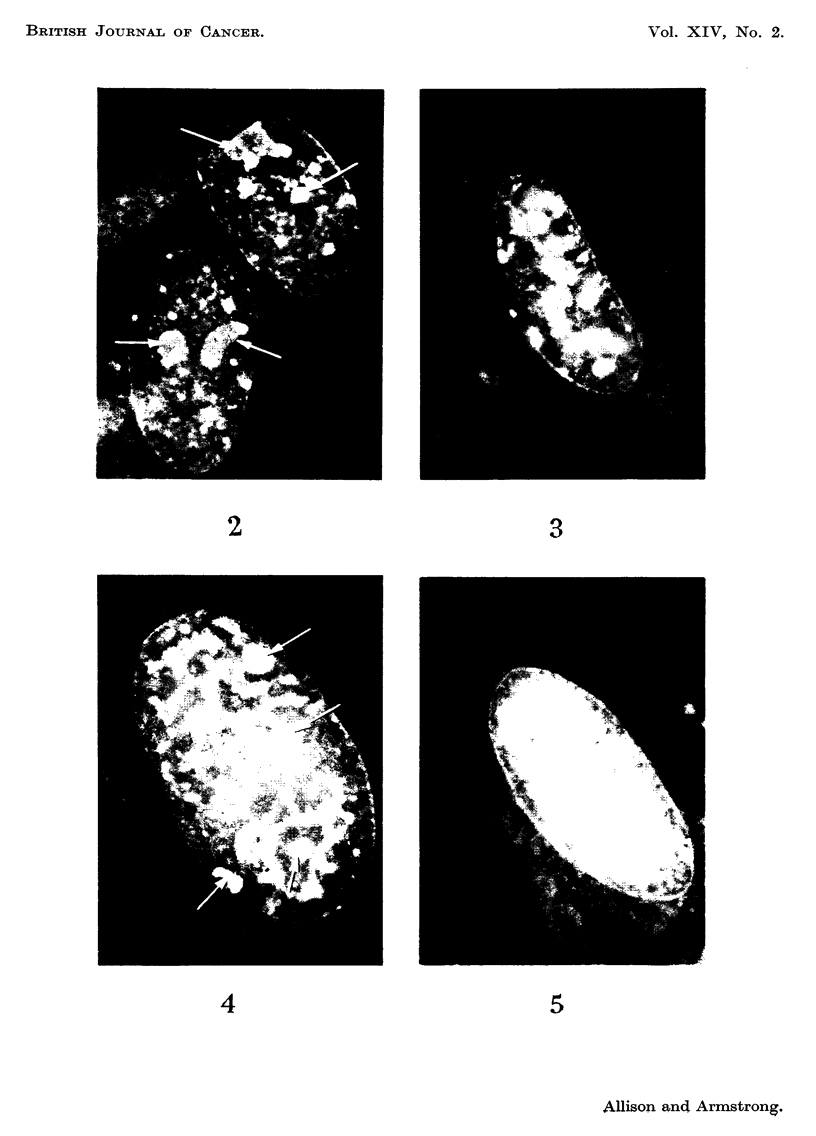

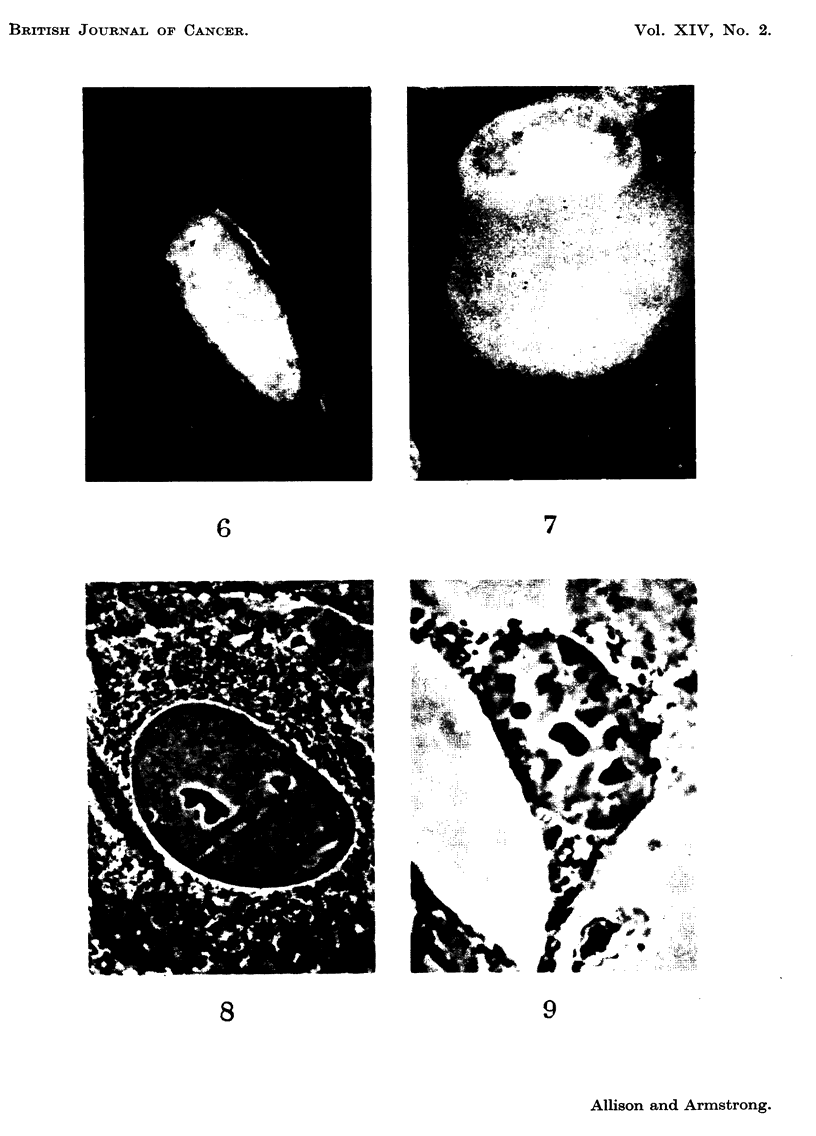

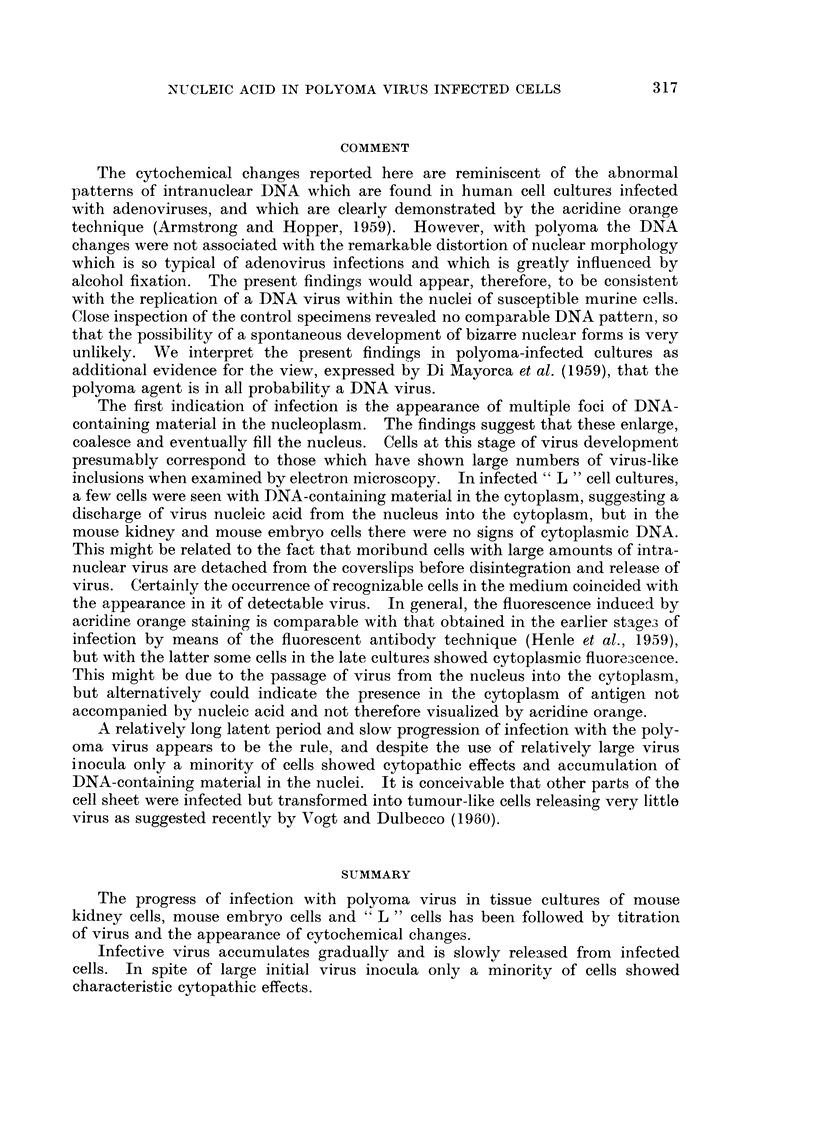

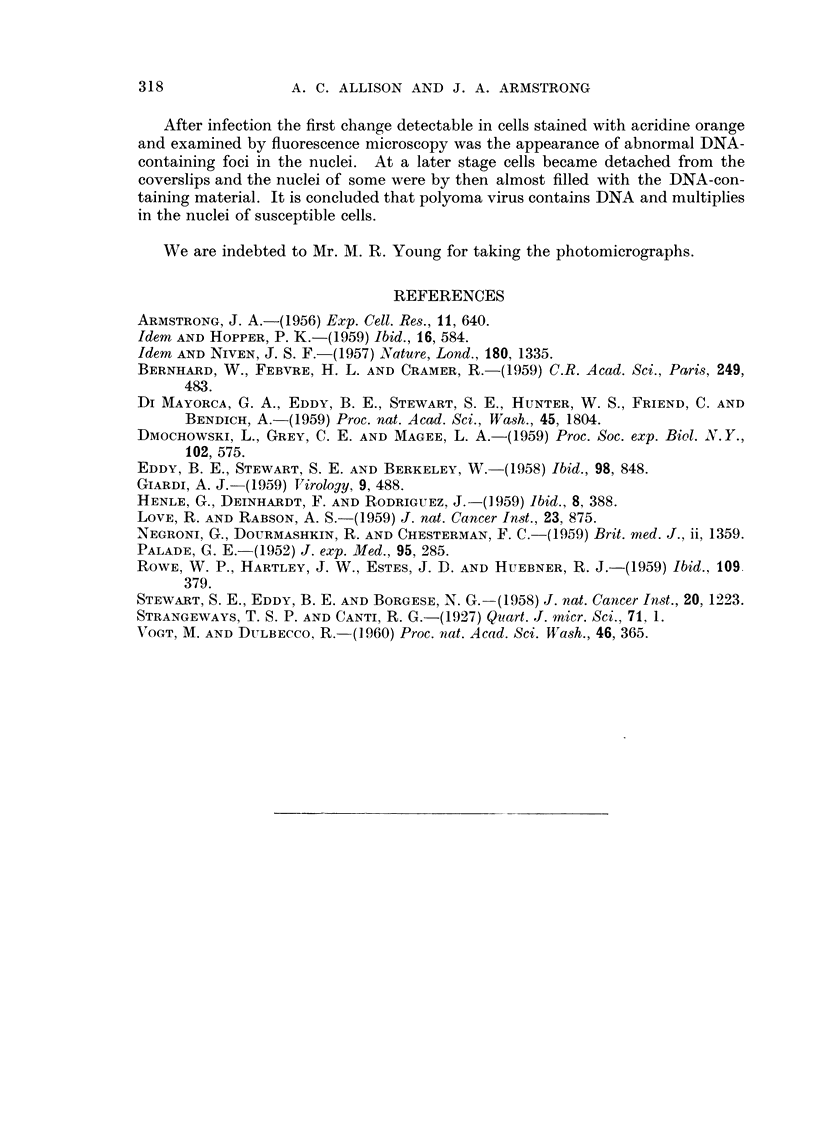


## References

[OCR_00337] ARMSTRONG J. A., NIVEN J. S. (1957). Fluorescence microscopy in the study of nucleic acids; histochemical observations on cellular and virus nucleic acids.. Nature.

[OCR_00347] DMOCHOWSKI L., GREY C. E., MAGEE L. A. (1959). Studies on a virus ("polyoma") inducing multiple tumors in animals.. Proc Soc Exp Biol Med.

[OCR_00350] EDDY B. E., STEWART S. E., BERKELEY W. (1958). Cytopathogenicity in tissue culture by a tumor virus from mice.. Proc Soc Exp Biol Med.

[OCR_00352] HENLE G., DEINHARDT F., RODRIGUEZ J. (1959). The development of polyoma virus in mouse embryo cells as revealed by fluorescent antibody staining.. Virology.

[OCR_00353] LOVE R., RABSON A. S. (1959). Cytochemical studies of milk-adapted murine lymphoma cells (strain P388 D1) infected with polyoma virus.. J Natl Cancer Inst.

[OCR_00355] NEGRONI G., DOURMASHKIN R., CHESTERMAN F. C. (1959). A "polyoma" virus derived from a mouse leukaemia.. Br Med J.

[OCR_00356] PALADE G. E. (1952). A study of fixation for electron microscopy.. J Exp Med.

[OCR_00362] STEWART S. E., EDDY B. E., BORGESE N. (1958). Neoplasms in mice inoculated with a tumor agent carried in tissue culture.. J Natl Cancer Inst.

